# Improvement of bread making quality by supplementation with a recombinant xylanase produced by *Pichia pastoris*

**DOI:** 10.1371/journal.pone.0192996

**Published:** 2018-02-26

**Authors:** Carolina Cândida de Queiroz Brito Cunha, Aline Rodrigues Gama, Lorena Cardoso Cintra, Luiz Artur Mendes Bataus, Cirano José Ulhoa

**Affiliations:** 1 Federal University of Goiás, Campus Samambaia, Goiânia, Goiás, Brazil; 2 University of Brasília, Campus Darcy Ribeiro, Distrito Federal, Brasília, Brazil; Korea University, REPUBLIC OF KOREA

## Abstract

Xylanases (EC 3.2.1.8) are hydrolytic enzymes, which randomly cleave the β-1,4-linked xylose residues from xylan. The synthetic gene *xynBS27* from *Streptomyces sp*. S27 was successfully cloned and expressed in *Pichia pastoris*. The full-length gene consists of 729 bp and encodes 243 amino acids including 51 residues of a putative signal peptide. This enzyme was purified in two steps and was shown to have a molecular weight of 20 kDa. The purified r-XynBS27 was active against beechwood xylan and oat spelt xylan as expected for GH 11 family. The optimum pH and temperature values for the enzyme were 6.0 and 75 °C, respectively. The *K*m and *V*max were 12.38 mg/mL and 13.68 μmol min/mg, respectively. The r-XynBS27 showed high xylose tolerance and was inhibited by some metal ions and by SDS. r-XynBS27 was employed as an additive in the bread making process. A decrease in firmness, stiffness and consistency, and improvements in specific volume and reducing sugar content were recorded.

## Introduction

Xylanases (EC 3.2.1.8) catalyze random endo-hydrolysis reactions of xylosidic links within the xylan chain to yield shorter xylooligosaccharides that are subsequently converted to xyloses by β-xylosidases (EC3.2.1.37) [1,2]. These enzymes have gained importance in biotechnology owing to their potential for application in various industries such as paper, animal feed, food manufacture, fermentation, and more recently, in biofuel production [3]. These biotechnological processes generally require harsh conditions and demand enzymes with specific properties, such as thermostability and thermotolerance [4].

Xylanase are produced by fungi, yeast, bacteria and actinomycetes [3–5] and show a broad range of substrate specificities. Actinomycetes are aerobic, gram-positive bacteria that have high GC content in their DNA. They form extensive branching substrates, and aerial mycelia with numerous pigmentations, and are widely distributed in soil [6]. Among Actinomycetes, *Streptomyces* spp. is dominant and is considered economically important in the production of commercial enzymes and secondary metabolites [3]. The advantages of xylanases produced by *Streptomyces* spp. include the high level of extracellular activity of these enzymes, their thermal stability (50–85 °C), and their stability across a broad pH range (pH 3–13) [3]. Xylanases from *Streptomyces* has been characterized, cloned and expressed in *Pichia pastoris* and *Escherichia coli* [7–12].

Bread has been the most common and traditional human food around the world for thousands of years [13]. In Brazil, baking is one of the fastest growing economic sectors, creating opportunities for development of new products based on enzymes [14]. Such enzymes as proteases, xylanases, and cellulases have gained importance in bread making [3,15]. Amylase and protease occupy the main positions in the market, but the demand for xylanases has increased in recent years. Xylanases are widely used as additives in the baking industry to improve processing and products quality [13]. They have been showed to effect dough characteristics such as stability, flexibility, extensibility, and coherency, by modifying the elasticity of gluten network. This results in better crumb structure, improvement of crumb porosity, firmness, texture profile, higher moisture retention and extend shelf life for bread [16]. Thus, it is important either to identify new microorganisms that produce enzymes with better performance, or develop efficient expression systems using already identified xylanases genes.

In this study, we describe the molecular cloning of a xylanase from *Streptomyces* sp. S27 and its expression in *Pichia pastoris*. We also describe the purification and characterization of this recombinant xylanase (r-XynS27) and its application in bread making.

## Materials and methods

### Strains, media and vectors

*Escherichia coli* TOP10 and the plasmid pGEM-T-Easy (Promega, Madison, WI, USA) were used for gene cloning. Transformants were selected on LB agar plate (10 g/L tryptone, 5 g/L NaCl, 5 g/L yeast extract, 15 g/L agar, pH 7.2) containing 50 μg/mL ampicilin (Promega, Madison, WI, USA).

*Pichia pastoris* protease-deficient strain SMD1168 (*his*4, *pep*4) and GS115 (*his*4) were used to produce recombinant protein using the expression vector pHIL-D2 (Invitrogen, Carlsbad, CA, USA). The culture media used were prepared as established by the *P*. *pastoris* expression kit from Invitrogen (Invitrogen, Carlsbad, CA, USA). Buffered Glycerol-Complex Medium–(BMGY-U) [100 mM potassium phosphate pH 6.0, 1% (w/v) yeast extract, 2% (w/v) peptone, 1.34% (w/v) urea, 4 x 10^−5^% (w/v) biotin and 1.0% (v/v) glycerol] and Buffered Methanol-Complex Media (BMMY-U) [100 mM potassium phosphate pH 5.0, 1% (w/v) yeast extract, 2% (w/v) peptone, 1.34% (w/v) urea, 4 x 10^−5^% (w/v) biotin, 1% (v/v) methanol and 50 μg/mL ampicilin] were prepared as described previously [17].

### Plasmid construction and expression of *xynBS27* in *P*. *pastoris*

A xylanase gene *xynBS27* (accession number EU660497) from *Streptomyces sp*. S27 was synthesized by Eurofins^®^ according to the codon usage for *P*. *pastoris*. The synthetic gene encoding the *XynBS27* was digested with *Eco*RI and subcloned into pHIL-D2 vector under control of alcohol oxidase 1 (AOX1) promoter. The recombinant pHIL-D2- *xynBS27* plasmid was linearized with *Sac*I and then transformed into *P*. *pastoris* GS115 and SMD1168 competent cells by electroporation using a Gene Pulser electroporator (Bio-Rad, Hercules, CA, USA).

Transformants cells were initially selected by the ability to grow on Minimal Dextrose (MD) agar plates [1.34% (w/v) Yeast Nitrogen Base (YNB) (Invitrogen, Carlsbad, CA, USA), 4 x 10^−5^% (w/v) biotin and 1.0% (w/v) dextrose] without histidine. The integration of expression cassette into the genome of pHIL-D2- *xynBS27* strains was verified by PCR using AOX5 (5’-GACTGGTTCCAATTGACAAGC-3’) and AOX3 (5’-GCAAATGGCATTCTGACATCC-3’) primers in accordance with the instructions of the *P*. *pastoris* expression kit (Invitrogen, Carlsbad, CA, USA).

Selected positive colonies were cultured in 1-L Erlenmeyer flasks containing 100 mL of BMGY-U medium at 28 °C under shaking at 200 rpm, until optical density at 600 nm reached 5–6. The cells were collected by centrifugation (12.000 x *g* for 15 min) and transferred to 1-L Erlenmeyer flasks containing 100 mL of BMMY-U medium, followed by incubation at 28 °C under shaking at 200 rpm for 7 days. To maintain induction, methanol was added every day to a final concentration in the range of 1 to 4% (v/v). After 7 days of induction, the cells were harvested by centrifugation at 12.000 x *g* for 15 min, and supernatant was used as source of xylanase.

### Xylanase activity assay

A standard xylanase activity assay was performed at 50 °C for 5 min in McIlvaine buffer, pH 6.0 (citric acid/disodium hydrogen phosphate) containing 1.0% (w/v) of beechwood xylan. The reducing sugars released by the reaction were measured at 510 nm using a microplate reader (ELx800, Biotek, Winooski, EUA), using the 3,5-dinitrosalicylic acid (DNS) method [18]. One unit (U) of xylanase activity was defined as the amount of enzyme that releases 1 μmol of reducing sugars from beechwood xylan per minute.

### Purification of r-XynBS27

An aliquot of 100 mL of culture filtrate was subjected to precipitation with 100% acetone, and centrifuged at 3.500 rpm, 5 °C, for 20 min. After discarding the supernatant, the pellet was resuspended with 50 mM phosphate buffer, pH 6.0, and submitted to ultrafiltration using an NMWL centrifugal filter with a 10 kDa cutoff (Millipore, USA). The concentrated sample (1 mL) was applied to a Sephadex G75 gel filtration column (1.5 × 28 cm), previously equilibrated with 50 mM phosphate buffer, pH 6.0. The proteins were eluted by washing the column with the same buffer at a flow rate of 12 mL/h. Fractions containing xylanase activity were pooled, dialyzed against water, lyophilized, and stored at −20 °C.

Protein concentrations were measured according to the Bradford method [19] using serum albumin as standard. In chromatography experiments, the protein contents of each fraction was estimated by measuring the absorbance at 280 nm. SDS-PAGE was used to determine protein purity and the molecular mass of the purified enzyme under denaturing conditions, using a 12% acrylamide gel, as described elsewhere [20]. Pierce^™^ Unstained Protein Molecular Weight Marker kit (Thermo Scientific) was used as a marker in the SDS-PAGE gel. The zymogram was performed as described previously [21], using 0.1% (w/v) beechwood xylan as a substrate.

### Biochemical characterization of purified r-XynBS27

The influence of pH on the enzyme activity was evaluated by varying the pH of the reaction mixtures using 50 mM McIlvaine buffer (pH 3.0–8.0) and 50mM Tris-HCl (pH 8.0–9.0). For the pH stability study, one volume of the enzyme was mixed with three volumes of each buffer and incubated at 4 °C for 12 h, after which the remaining xylanase activity was measured at pH 6.0 and 50 °C.

The effect of temperature on the enzymatic activity was investigated at pH 6.0, over a range of 30 °C to 80 °C. Thermostability was measured by pre-incubating the enzyme at 50, 60 and 70 °C for 360 min. The effects of metal ions, chelating agents, or surfactant solutions on the r-XynBS27 activity were determined after the enzyme had been incubated with the reagents at final concentration of 5 mM and pH 6.0. The effect of xylose on r-XynBS27 activity was evaluated after preincubation of the enzyme with different concentrations (40, 80 and 160 mM) of these compounds as described elsewhere [22]. After 60 min incubation at 50°C, the activity of the r-XynBS27 was determined using beechwood xylan as substrate.

The Km and Vmax values for r-XynS27 were calculated from a Lineweaver–Burk plot. The xylanase activity was assayed at 60 °C in McIlvaine buffer, pH 6.0, containing 2.5–25 mg/mL beechwood xylan as the substrate. Each experiment was repeated three times and each experiment included three replicates.

### Effects of addition of r-XynBS27 on bread production

The bread dough was formulated with wheat flour (435 g), dried yeast (5 g), salt (3 g), sugar (60 g), oil (30 mL), egg (1 unit) and milk (200 mL). r-XynBS27 was added at concentrations of 75, 150 and 300 U/Kg of flour. The enzyme was dissolved in milk before be used during the initial preparations of bread dough to ensure its broad distribution throughout the mass during the mixing process. The mixture was mixed for 1 min in a domestic bakery machine (Britânia, Brazil). During the manufacture of bread, 1 g was removed before baking for analysis of reducing sugar production [18] by the enzyme in the different formulations. The bread dough was baked at 180°C for 25 min.

The volume of bread baked was determined after 24 hours by rapeseed displacement method as described by AACC (2000). The specific volume was calculated by dividing the bread volume (mL) by its mass (g). After 24 h storage, crumb firmness, consistency and stiffness were measured using a TA-XT2i texturometer (Stable Micro System, England). Selected bread slices were cut (5 cm x 5 cm) and analyzed immediately for investigation of bread firmness, stiffness and consistency. A cylinder probe of 10 mm diameter was attached to a moving cross-head. Samples were subjected to a double cycle of compression under the following conditions: 5 mm/s cross-head speed and 40% maximum deformation. The maximum force (Fmax) needed to deform each cube was recorded and is referred to as crumb firmness. The texture profile parameters were evaluated using the Texture Expert 1.22 software (Stable Micro Systems).

All the measurements were conducted in triplicate. The significance level (p-value) of each concentration effect was evaluated by applying the Student’s t-test using Statgraphics ^®^ software.

## Results and discussion

### Expression of r-XynB27gene in *P*. *pastoris*

To identify xylanase genes encoding candidate enzymes for application in baking, we performed a search on GenBank (http://www.ncbi.nlm.nih.gov/genbank/). The xylanase gene *xynBS27* (accession number EU660497) from *Streptomyces sp*. S27 was chosen due the enzyme presenting activity over broad pH range (4.0–8.0), good thermostability at 60 °C and being efficient in releasing oligosaccharides from xylan [9]. The synthetic gene encoding the *xynBS27* was subcloned into pHIL-D2 vector and used to transform *P*. *pastoris* GS115 and SMD1168 strains. The *P*. *pastoris* SMD1168, was used because it is a proteinase A-deficient strain [23]. Several xylanases have been successfully produced by heterologous expression in the yeast *P*. *pastoris* [24].

Transformants generated by electroporation experiments with recombinant plasmid (pHIL-D2/*xynBS27*) were first selected on MD agar plates without histidine and subsequently grown in BMMY-U liquid medium. Approximately 280 transformants were screened, 52 from GS115 and 223 from SMD1168. Nineteen transformants (being 47 from GS115 e 94 from SMD1168) were screened for xylanase activity of the culture supernatant after growth in deep well plate containing BMMY-U liquid medium. Most of the clones showed xylanase activity, but two (*xynBS27*/GS115 and *xynBS27*/SMD1168) that exhibited higher activity were selected for further studies. None of these clones showed activity avicel (1%), CM-cellulose (4%), filter paper (8mg), p-NPA (5mM), p-NPG (6mM) and p-NPX (7mM).

**Fig 1:**
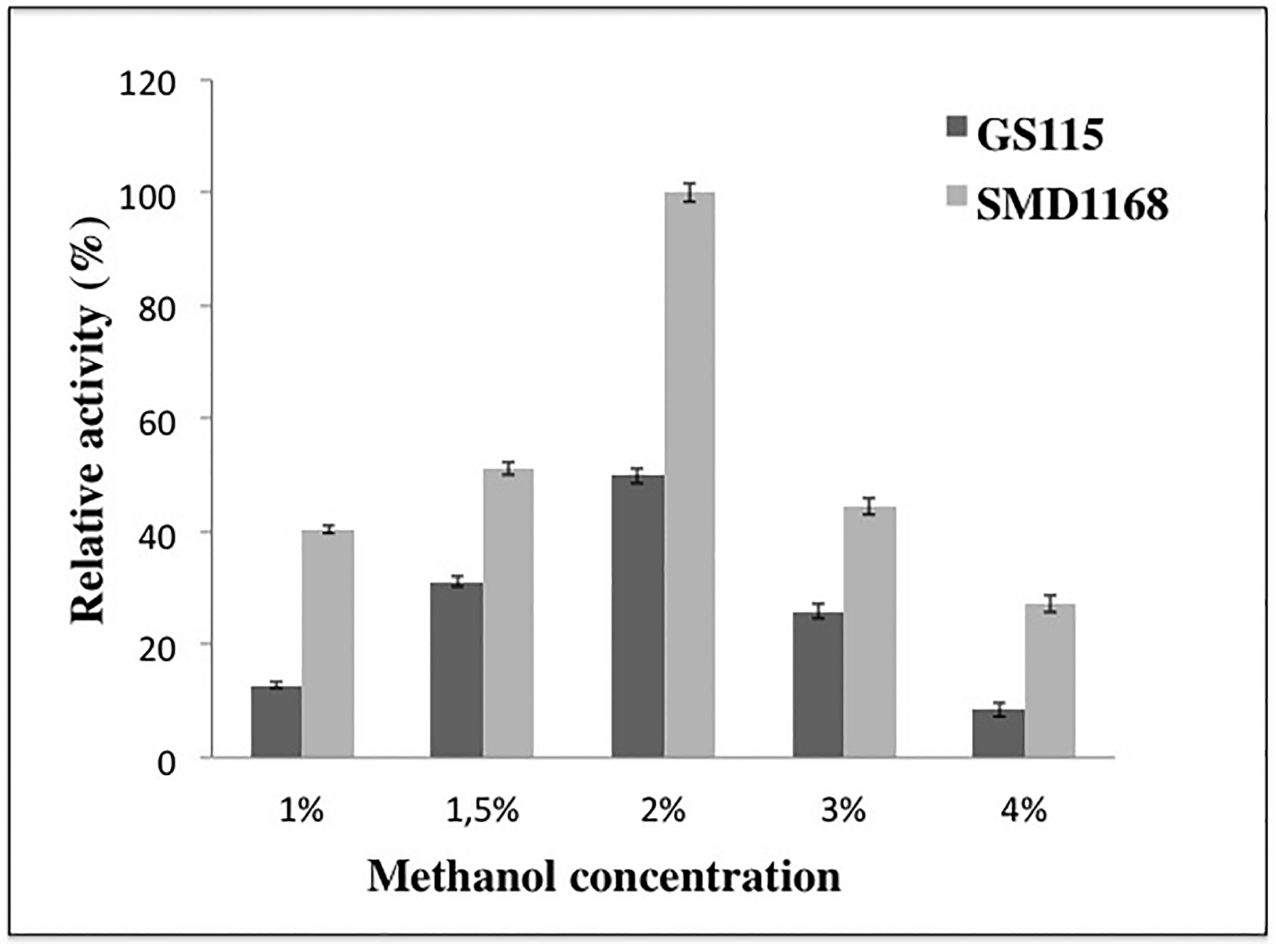
Comparative studies comparatives of xylanase productions by *P*. *pastoris* GS115 and SMD1168 strains, varying the concentration of methanol.

Both transformants were inoculated in 250 mL erlenmeyer flasks together with 50 mL of BMMY-U liquid medium supplemented with different methanol concentrations (1.0 to 4.0%). In the *P*. *pastoris* expression system, methanol is used as the carbon source and as the inducer for protein expression. The optimal concentration of methanol in the production of xylanase by *xynBS27*/GS115 (42 U/mL) and *xynBS27*/SMD1168 (78.7 U/mL) was 2.0%, after incubation for 96 hours at 28 °C (Fig 1).

**Fig 2:**
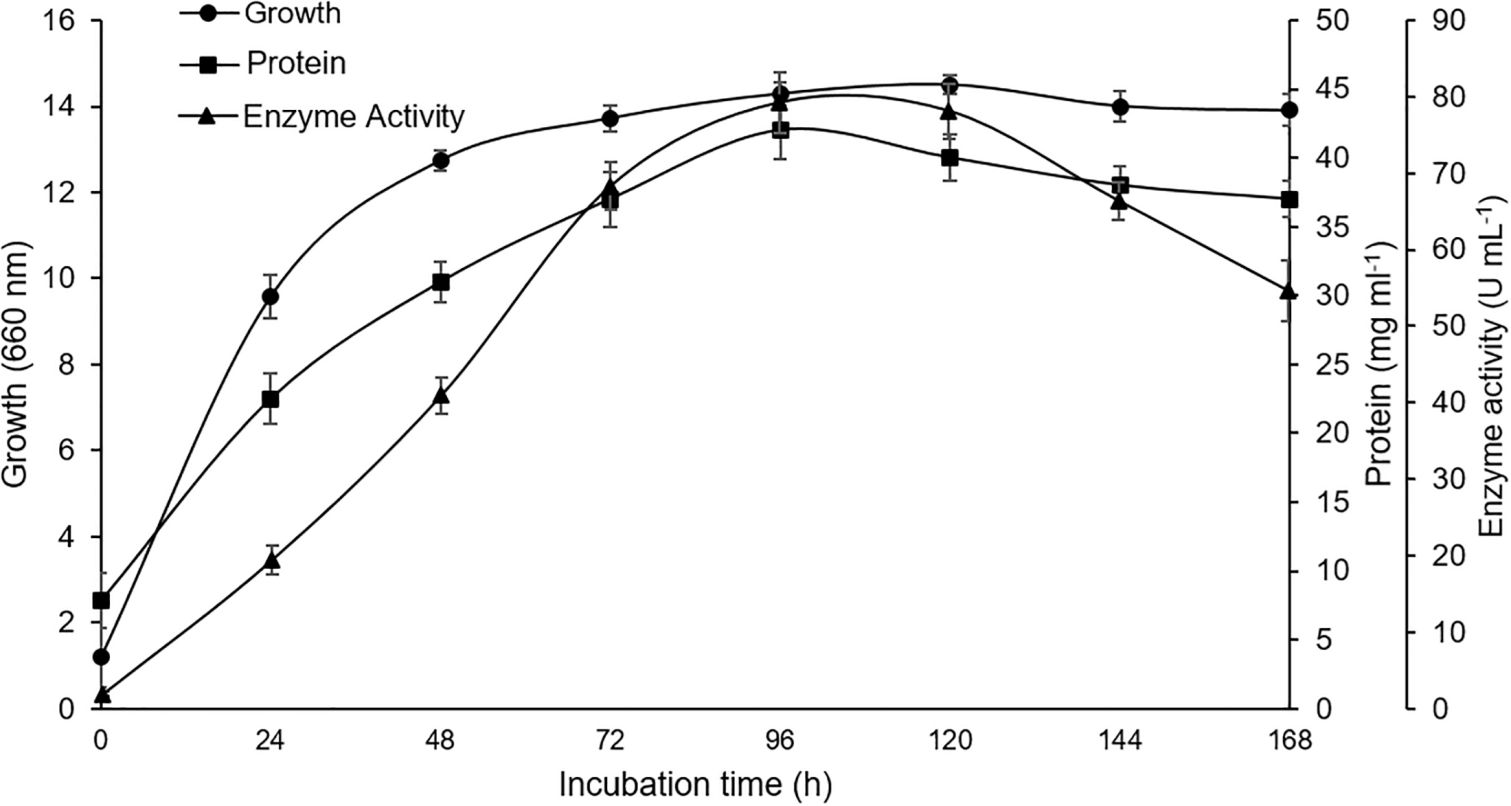
Time course of growth (triangle), protein (circle) and xylanase production (square) by *P*. *pastoris* SMD1168.

Several studies have reported that higher methanol (>2.0%) concentrations are better for cell growth rate and improved protein yield [25]. However, negative effects of high methanol concentration on productivity also have been reported [26]. Low concentrations of methanol (< 1.0%) may result in insufficient transcription and high concentrations (> 2.0%) could be toxic to the cells. The clone that showed the highest activity was from SMD1168 strain, and for this was chosen for enzyme production on a larger scale (Fig 1). The time course of xylanase and protein production by the *xynBS27*/SMD1168 on BMMY-U liquid medium supplemented with 2.0% (v/v) methanol is shown in Fig 2.

Protein production (mg/mL) was accompanied by cell growth (600 nm) and xylanase activity (U/mL). Extracellular xylanase activity increased during cell growth and reached a maximum value (82 U/mL) at 96 h of incubation, with activity decreasing slowly thereafter. The supernatant produced by *xynBS27*/SMD1168 was used in purification of recombinant xylanase (r-XynB27).

### Purification of recombinant r-XynB27

We purified a r-XynS27 secreted by *xynBS27*/SMD1168, after growth in BMMY-U liquid medium, by using acetone precipitation and gel filtration on Sephadex G75 (Table 1). The r-XynS27 was purified 2.90-fold with a recovery of 14%. In most published reports of xylanase purification from bacteria, two or more steps of chromatography have been used [3]. For example, a xylanase from *Streptomyces althioticus* LMZM was purified by a combination of ammonium sulphate precipitation, Sephadex G-25, DEAE cellulose chromatography, followed by gel filtration through a Sephadex G-100 column [27].

**Table 1:** Summary of the purification steps of the xylanases (r-XynS27) produced by *P*. *pastoris*.

Purification Steps	Enzyme Activity (U/mL)	Protein (mg/mL)	Specific Activity (U/mg)	Yield (%)	Purification (fold)
Crude enzyme	24.2	12.4	1.95	100	1
Acetone precipitation	22.0	9.6	2.29	91	1.17
Sephadex G75	3.4	0.6	5.67	14	2.90

**Fig 3:**
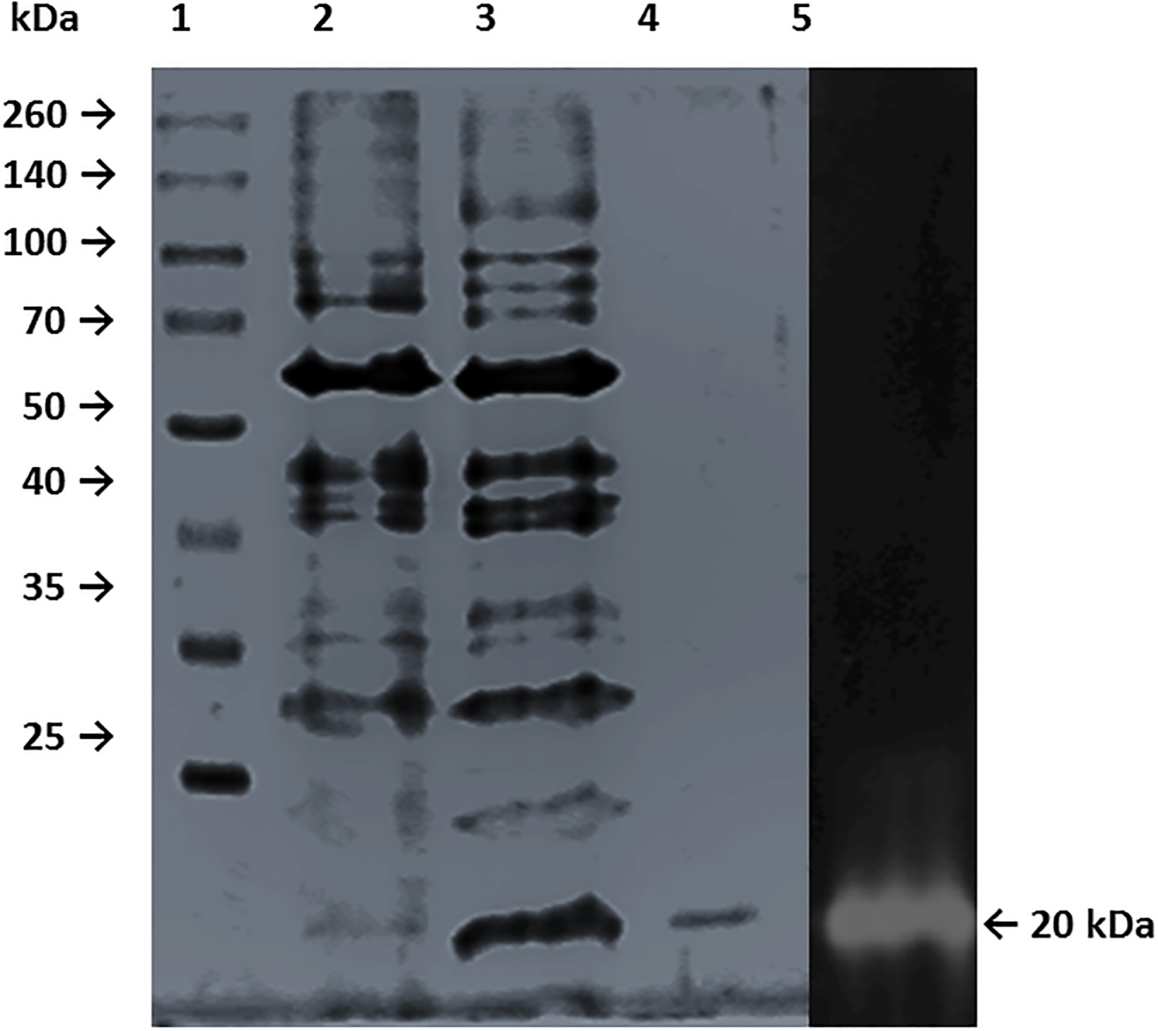
SDS-PAGE analysis of purified r-XynS27. Lane 1, molecular weight markers; lane 2, crude enzyme; lane 3, Acetone precipitation; lane 4, Sephadex G75 fraction; lane 5, zymogram of purified r-XynS27.

The purified r-XynS27 protein appeared as a single band on SDS-PAGE with a molecular mass of 20 kDa (Fig 3) and was similar to the xylanases from *Streptomyces matensis* [28]. However, xylanases from *Streptomyces sp*. SWU10 (31 and 44 kDa), *Streptomyces thermovulgaris* TISTR1948 (46.2 kDa), *Streptomyces* sp. CS624 (40 kDa), *Streptomyces* sp. CS428 (37 kDa) and *Streptomyces* sp. FA1 (43 kDa) showed greater larger molecular weight than r-XynS27 [11,12,29,30].

In order to show that the protein band with 20 kDa corresponds to a xylanase we performed a zymogram adding beechwood xylan into SDS-PAGE. After staining with Congo red, substrate hydrolysis, corresponding to r-XynS27activity, appears as clear zones against a black background (Fig 3, lane 5).

### Biochemical characterization of purified recombinant r-XynS27

**Fig 4:**
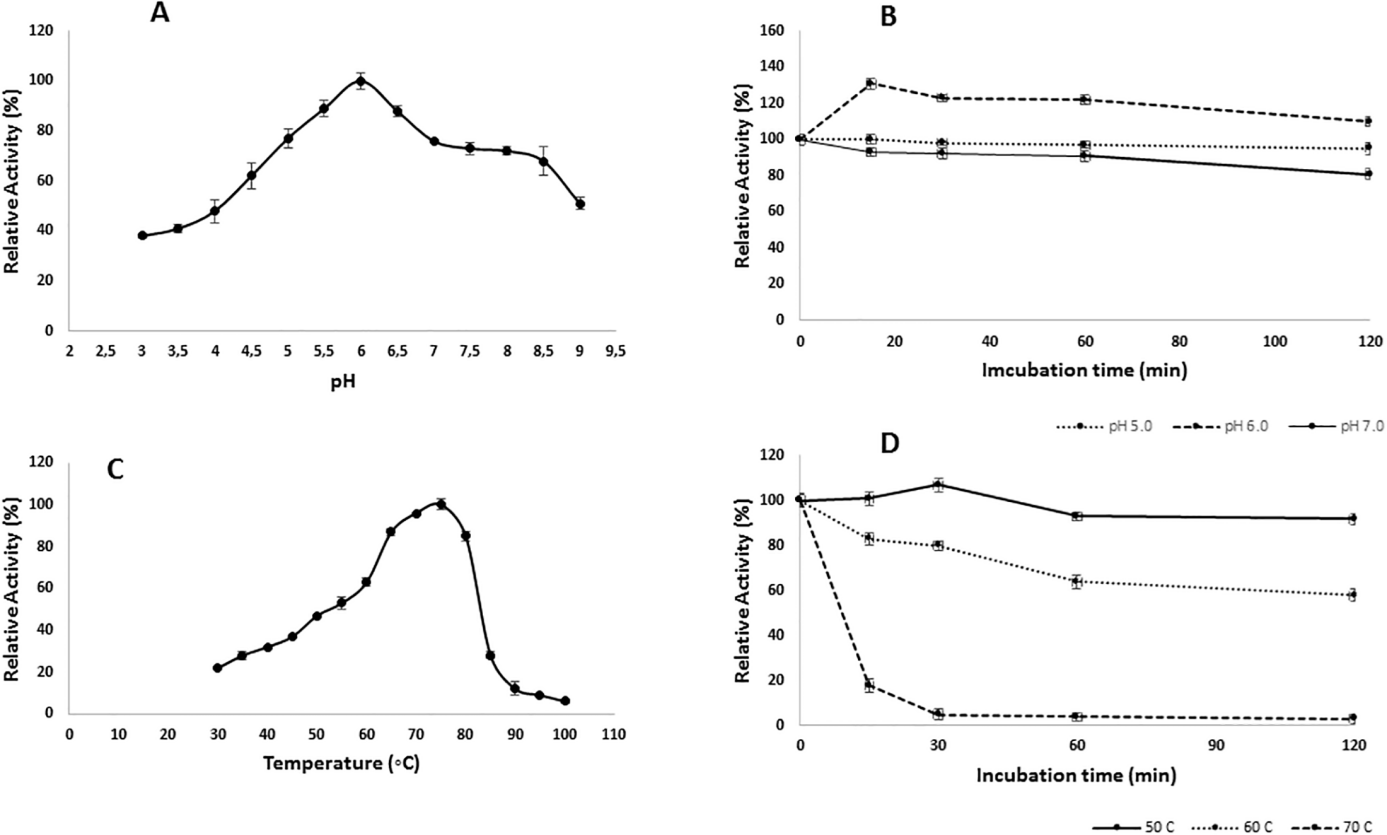
A) Effect of pH on r-XynS27 activity; B) Effect of pH on r-XynS27 stability; C) Effect of temperature on r-XynS27 activity; D) Effect of temperature on r-XynS27stability. The data given are the mean of two independent experiments. The bars represent the standard deviation.

The optimum pH value for the purified r-XynS27 was found to be 6.0, but the enzyme keep 60% of its initial activity at pH 4.5 and 8.5 (Fig 4A).

Most of the previously reported *Streptomyces* xylanases have optimal pH values in the range of 5.0 to 7.0 [3]. For example, the purified xylanases from *S*. *megasporus* DSM 41476 [10], *Streptomyces* sp.SWU10 [11], *Streptomyces* sp.S9 [8] and *S*. *matensis* [28] showed the optimum pH levels of 5.5, 6.0, 6.5 and 7.0, respectively. pH stability studies with purified r-XynBS27 showed 80–100% retention of enzyme activity across pH 5.0 to 7.0 after incubation at 50°C for 120 min (Fig 4B). These wide ranges of pH activity and stability are important characteristics for industrial and biotechnological applications of these enzymes [3,5,31,32].

The influence of temperature on r-XynS27 activity was analyzed using a temperature range from 30 to 90 °C at pH 6.0 (Fig 4C). The optimum temperature for the r-XynS27 was 75°C (Fig 4C). 60% and 80% of the initial enzyme activity was retained at 60°C and 80°C, respectively. The optimal temperatures for the majority of previously described *Streptomyces* xylanases are between 60 and 70 °C [3,11,30,32]. However, *Streptomyces* sp. CS428 showed a xylanase with optimum temperature at 80 °C, and has been used to xylooligosaccharides production [29]. r-XynS27 thermal stability was examined after incubation of the enzyme at 50, 60 and 70 °C. r-XynS27 showed complete stability at 50 °C, kept 60% of initial activity at 60 °C, and completely lost its activity after incubation at 70 °C for 120 min (Fig 4D). Although most xylanases produced by *Streptomyce*s sp. have an optimal temperature between 60 and 70°C, in general they lose activity rapidly when incubated for longer times at the optimal temperatures [5].

The influence of various xylose concentrations (40, 80 and 160 mM) on r-XynS27 activity was investigated (Table 2). The enzyme showed 40% inhibition by 160 mM xylose, suggesting a high level of resistance to xylose inhibition. These are interesting data since xylanases are essential enzymes in the conversion of xylan and xylooligosaccharides to xylobiose, which is further hydrolyzed to xylose by β-xylosidases [3,15].

**Table 2:** Effect of several metal ions, chemical compounds and xylose on r-XynS27.

Compounds	Relative Activity (%)
Control	100 ± 2.1
AlCl_3_	113 ± 2.3
NH_4_Cl	142 ± 2.8
BaCl_2_	147 ± 2.8
CaCl_2_	99 ± 3.0
CuCl	72 ± 4.0
LiCl	101 ± 3.1
MgCl_2_	135 ± 3.0
HgCl	34 ± 1.4
AgCl	0 ± 2.1
NaCl	98 ± 3.0
KCl	90 ± 1.5
MnSO_4_	54 ± 1.7
ZnSO_4_	67 ± 2.1
FeSO_4_	63 ± 2.3
β-mercaptoetanol	152 ± 2.3
EDTA	91 ± 2.0
SDS	51 ± 2.0
Xylose 40 mM	81 ± 0.6
Xylose 80 mM	69 ± 0.9
Xylose 160 mM	60 ± 0.5

The influences of metal ions and chemical reagents on xylanase activity were also examined (Table 2). Activity of r-XynS27 was increased by β-mercaptoethanol, was not affected by Ca^2+^, was weakly inhibited by EDTA, and strongly inhibited by Hg^2+^ and SDS. The capacity of β-mercaptoethanol to activate the enzyme suggests that cysteine residues are present in the active site of the enzymes. Most xylanases are inhibited by Hg^2+^, suggesting the presence of cysteine thiol groups in their active sites [33]. SDS strongly inhibited enzyme activity, indicating the likely importance of hydrophobic interactions in maintaining enzyme structure. Enzyme activity was almost completely retained the presence of NaCl (Table 2). Salt tolerance is an important characteristic, since NaCl is an important component of bread dough formulation [34].

Kinetic parameters of r-XynS27 were examined by incubating the enzyme with increasing concentration of beechwood xylan (2.5–25 mg/mL) at 50 °C, pH 6.0. These parameters were established using a Lineweaver–Burk plot. The Km and Vmax values were estimated to be 12.38 mg/mL and 13.68 μmol/min/mL, respectively. Compared with other xylanases assayed with beechwood xylan, the *K*_m_ value of r-XynS27 was lower than that from *Streptomyces* sp. CS428 (102.30 mg/mL), but higher than that from *Streptomyces sp*. FA1 (3.45 mg/mL), *Streptomyces* sp. CS624 (5.61 mg/mL) and *Streptomyces thermovulgaris* TISTR194 (0.76 mg/mL) [29,30,32]. However, comparisons between Km of xylanases are difficult since measurements rarely share the same conditions such as: incubation time, temperature, heating rate, protein concentration, and mainly substrate nature and concentration [35].

### Effects of addition of r-XynBS27 on bread production

Xylanases have been used in bread making to improve dough and bread properties [3,15]. However, these enzymes have not been applied extensively in baking industry because of high cost and lower stability during the baking process. Thus, it is highly desirable to adopt gene engineering to produce enzymes with better performance and lower cost. A xylanase from *Sreptomyces* S27 produced by *P*. *pastoris* (r-XynS27) showed interesting characteristics for use in baking process such as high stability at pH 5.0–7.0, thermostability at 50–60 °C, and high degree of tolerance to xylose inhibition.

To test the performance r-XynS27 in bread making, a comparative study of bread attributes after xylanase supplementation was performed (Table 3).

**Table 3:** Effect of r-XynS7 in some characteristics of bread prepared by different treatment of enzymes.

Attributes	Control	75 U/Kg	150 U/Kg	300 U/Kg
Reducing sugar (mg/mL)	2.65±0.01 -	3.28±0.02* (+24%)	3.60±0.10* (+36%)	4.19±0.07* (+58%)
Volume (cm^3^)	2.82±0.18 -	3.30±0.20* (+17%)	3.50±0.20* (+24%)	3.20±0.20* (+13%)
Density (g/cm^3^)	261±10.6 -	219±9.5* (-16%)	217±5.5* (-17%)	223±5.9* (-15%)
Specific volume (cm^3^/mg)	3.83±0.24 -	4.55±0.28* (+18%)	4.57±0.26* (+19%)	4.47±0.28* (+17%)
Water loss (g)	4.10±1.50 -	2.70±1.25* (-66%)	2.00±0.90* (-49%)	2.60±1.20* (-63%)
Firmness (N)	14.19±1.1 -	4.97±0.90* (-65%)	5.07±0.80* (-64%)	5.04±0.80* (-65%)
Consistency (Kgf/mm)	9.80±0.3 -	2.92±0.10* (-70%)	3.08±0.08* (-69%)	3.11±0.10* (-68%)
Stiffness (N/mm)	9.70±0.5 -	0.60±0.03* (-94%)	0.53±0.09* (-95%)	0.93±0.04* (-90%)

*Significantly different from the control group at p<0.05. Data represent mean ± standard deviation of three replicates. The values in parenthesis represent the increase (+) or decrease (-) of the attributes.

We choose to test three different enzymes concentrations (7.5, 15 and 30 IU/100 g of flour) according to studies described in the literature concerning the application of microbial xylanases in the bread making [3,15]. Initially, the effects of r-XynS27 on the liberation of reducing sugars during the preparation of the dough were analyzed. All enzymatic treatments showed a significant increase in reducing sugars in dough, when compared with the control without enzymes (*p* < 0.05, Table 3). Increases of 24%, 36% and 58% of reducing sugars in the dough were observed at enzyme concentration of 75, 150 and 300 U/ Kg of flour, respectively. The results showed that r-Xyn27 was effective in releasing of reducing sugar such as xylobiose, xylotriose and xylotetraose from hemicelluloses present in wheat flour. These reducing sugars might be used by yeast for growth, fermentation and improvement of gas-retention as described by other xylanases used in bread making [3,15,36,37].

**Fig 5:**
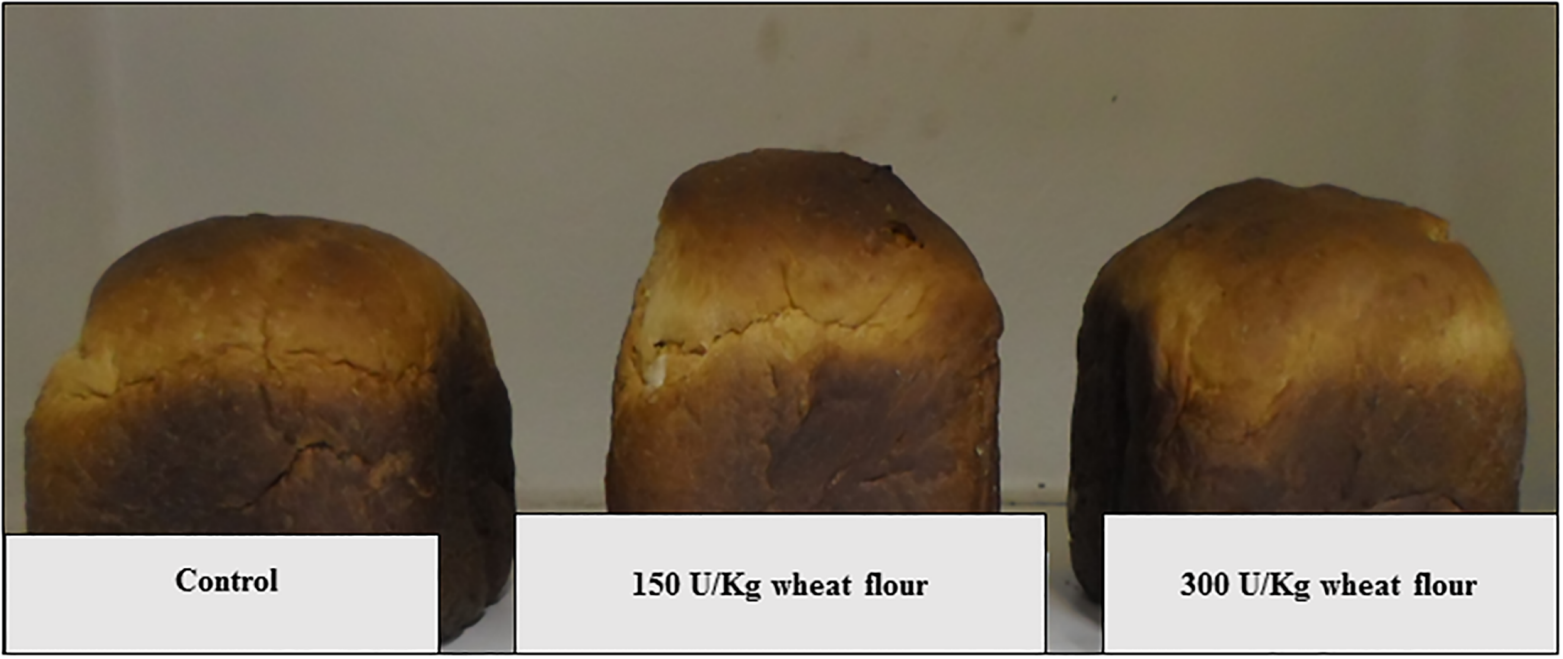
Effects of different concentrations of r-XynS27 on bread volume.

We observed a significant increase in bread volume in all enzymatic treatments, when compared with the control (*p* < 0.05, Table 3, Fig 5).

Consequently, a decrease in the density and an improvement in the specific volume of the loaves were also observed (*p* < 0.05, Table 3). Specific volume, which involves loaf volume and loaf weight, is an important indicator of the quality of bread [15]. The addition of r-XynS27 resulted in softer bread, since the values of firmness, consistency and stiffness were significantly lower than the control (*P* < 0.05, Table 3). Improvements in crumb firmness can be explained as a result of the increase in loaf volume. Breads with low specific volumes have a disagreeable appearance and are associated with high moisture content, failure in cooking, poor aeration, difficult chewing, improper taste and low conservation.

The positive or negative influence of xylanase on the specific volume of the loaves is related to the quantity and characteristics of arabinoxylan present in the wheat flour [38]. Cereal arabinoxylan (AX) is classified into water-extractable arabinoxylan (WE-AX) and water-unextractable arabinoxylan (WU-AX) [39]. The WU-AX can negatively affect the formation of gluten because of interference during interactions of gliadin and glutenin proteins [40]. A xylanase that degrades WU-AX, such r-XynS27, could help in the formation of a more flexible and stable dough that would have a greater ability to expand during baking.

## Conclusion

In the present study, a xylanase (XynS27) from *Streptomyces* S27 was cloned and expressed in *P*. *pastoris* SMD1168. The enzyme was purified and showed interesting characteristics for use in baking process, such as high stability at pH 5.0–7.0, thermostability at 50–60 °C and high tolerance to xylose inhibition. This purified r-XynS27 was used in bread making and showed to effective in improvements of some characteristics of the bread. All enzymatic treatments showed a significant increase in reducing sugars in dough favoring the fermentation and gas-retention. A significant increase in bread volume, decrease in the density and an improvement in the specific volume were observed. Firmness, crunchiness and stiffness were significantly lower comparing with the control, resulting in softer bread.
